# DrugPred: an EdgeConv-GNN and Bio_ClinicalBERT based polypharmacy ADR prediction and specialist recommendation model

**DOI:** 10.3389/frai.2026.1874825

**Published:** 2026-07-29

**Authors:** J. G. Arjay, K. Adit Pushan, N. Roshanthraj, Brindha Subburaj, Sherly Alphonse, Girish Subramanian

**Affiliations:** 1 School of Computer Science and Engineering, Vellore Institute of Technology, Chennai, India; 2 School of Business Administration, Penn State Harrisburg, Middletown, PA, United States

**Keywords:** adverse drug reaction prediction, clinical decision support system, pharmacovigilance, polypharmacy, public health

## Abstract

Adverse drug reactions (ADRs) are caused by medication and are considered a serious issue in healthcare when there is simultaneous use of different medications resulting in drug-drug interaction (DDI). Traditional approaches mostly focus on the effects caused by a single drug, and they fail to capture the side effects from drug combinations. In this research work, a deep learning-based DrugPred framework is proposed to predict ADR risks by integrating individual drug effects, interaction statistics of the drugs, and structural relationships between drug-interaction graphs. In the first phase, baseline association scores between individual drugs and their corresponding ADRs, such as nausea, liver toxicity, or cardiovascular effects, are learned using a multi-layer perceptron (MLP) trained on the OFFSIDES dataset, providing probabilistic values for individual drug-ADR pairs. In the second phase, a Graph Neural Network (GNN) based on the EdgeConv architecture is utilized to model drug-drug interactions using the TwoSIDES dataset, capturing relational dependencies by utilizing pairwise statistical values such as co-occurrence frequency and interaction statistics derived from pharmacovigilance signals, including transformed PRR-based signals. In the third phase, Bio_ClinicalBERT, which is a pre-trained transformer-based language model specifically trained on clinical notes and biomedical text, is used to encode drug pair representations and combine them with association and interaction scores through an attention-guided mechanism, which enables the model to adaptively weight heterogeneous features and finally performs multi-label ADR prediction. The proposed model achieves an accuracy of 95.73%, F1-score of 0.94, ROC-AUC of 0.993, and PR-AUC of 0.988. These results indicate that our proposed DrugPred framework is effective in the prediction of ADR risks with high precision and recall. Overall, the proposed framework provides a scalable approach for predicting ADRs in multi-drug settings. We further extend our framework with a retrieval-based guidance system that maps the risk levels of the predicted ADRs to appropriate System Organ Classes (SOC) and medical specialists to provide recommendations using real-world clinical data sources like PubMed and OpenFDA.

## 1 Introduction

Clinical Decision Support Systems (CDSS) are increasingly using data-driven methods to help providers determine how best to treat their patients. An increasing volume of Electronic Health Records (EHRs) and patient-generated data has led to a strong need for personalized recommendation systems that cannot be supported by classic rule-based procedures (Dongre and Agrawal, 2023). Drug-Drug Interaction (DDI) is the interaction of two or more drugs when prescribed to patients by physicians. It is becoming an integral entity in the operation of CDSS (Pham et al., 2024). In most cases nowadays, modern intelligent recommender systems do not include DDI as part of their models in order to maintain efficiency in treatment for patients who are prescribed drugs by multiple providers. There are many studies that have explored existing recommender systems in the health care space, most frequently through the lens of collaborative filtering, content-based, and hybrid methods, with the purpose of supporting the diagnosis process as well as selecting treatment for patients. Many of these systems attempt to find the best-matching patient to the best treatment or medication for that patient. However, with regard to handling safety issues, these systems primarily rely on simple *post-hoc* checks, which significantly decreases the efficacy of these systems in the context of high-risk polypharmacy.

Recent developments in GNNs have helped to reshape the way we predict drug-drug interactions (DDIs). AutoDDI provides a GNN-based system to learn representations of drugs and patterns of drug interactions automatically, directly from a heterogeneous biomedical network. AutoDDI has demonstrated excellent predictive performance relating to the identification of DDIs (Gao et al., 2024). This work shows that the combination of DDI prediction through GNNs with social media-related ADRs and recommendation systems could provide a strong basis for safety-aware clinical decision support systems.

In a recent work, the Variation-Inferred Drug Target Association (ViDTA) framework is presented by Li et al. (2024), which advances drug-target affinity prediction through the use of virtual graph nodes and a feature fusion mechanism driven by attention. The Prior-Guided Graph Dual Contrastive Learning (PGDCL-DTI) framework, Liu et al. (2024), employs parallel dual-channel graph contrastive learning to extract complex structural features. Such an approach gave insights into how one can improve the model speed while dealing with graphs and also helped in contrasting and identifying high-confidence adverse reactions and background noise, ensuring that the predicted signals are different compared to the learned priors. Using GNN methods, the system learned interaction-aware embeddings that helped analyze how drug pairing increased the risks.

Our study's primary objective is to address the potential dangers that could arise from unsafe medication combinations while delivering tailored treatment recommendations. The research objectives are:

- To develop a framework to evaluate risks pertaining to the dangers of multiple drug usage at a given time. This helps enable drug reactions that would otherwise not be possible in traditional pharmacology practices.
- To connect historical medical pairs with already known data to map the findings and compare them with the ground reality (data from OFFSIDES taken as the ground reality for comparison and eventual data extraction from the users).
- A well-designed GNN framework with nodes and edges that learns the properties of different drugs interacting with each other, rather than just individual drug properties, thus incorporating multi-drug interactions.
- To create an end-to-end system that enables users to reach out to specific specialists who may cater to the needs of such adverse reactions to the drugs and thus provide specialist recommendations.

The research dives into an ADR-aware clinical decision support system that integrates medical records of a patient and predicts what ADRs the patient is expected to experience based on their drug usage and medical history. Moreover, with the predicted ADR, we formulated a clinical decision support system that guides the patient to consult the appropriate medical specialist for the ADR they are supposed to experience.

The framework is modeled to first derive the association scores for each drug-ADR pair, which is the probability of an ADR occurring based on a single drug using the OFFSIDES database by Tatonetti et al. (2012). The association score for each drug is used in the Drug-Drug interaction modeling, where we formed a graph consisting of nodes being each drug and edges representing the statistical information of each drug and the other drug it's related to, and with this graph, we used a GNN to form interaction scores for each drug-drug-ADR pair, which gives the likelihood of a particular ADR to occur based on multiple drugs administered to the patient. An attention gate mechanism then adaptively fuses the numerical pharmacovigilance signals as embedded clinical text. This resulting representation is passed through the fully connected layers with a sigmoid activation to create a multi-label probability vector that indicates the probability likelihood of occurrence of each of the 10 predefined ADRs for the drug pair evaluated. With the predicted ADRs and risk scores, the development of a clinical decision support system was accomplished by mapping each high ADR risk to the corresponding System Organ Class (SOC) and specialist, searching for evidence in OpenFDA Kass-Hout et al. (2016) and PubMed Fiorini et al. (2018) using Facebook AI Similarity Search (FAISS), and creating a structured referral recommendation for a specialist based on real-world clinical evidence.

## 2 Related works

This section dives into the literature study and the existing work in the field of Drug-ADR prediction. The subsections explain the individual components of the research objective and its background.

### 2.1 Traditional methods for ADR signal detection

ADRs are unwanted reactions and side effects to drugs that can range from having little to no effect on the person taking them to being very severe, even potentially fatal. In the pharmacovigilance field, many studies and research are conducted to study the connection and relation of an adverse event experienced by a person and the prescription of the drug the person is taking at that time.

Traditional methods to detect ADR signals were mainly statistical and rule-based methods, which were first established before the introduction of deep learning and model-based prediction. Reporting Odds Ratio (ROR) Rothman et al. (2004), which checks if a side effect is reported unusually for a particular drug compared to other drugs in the database. Bayesian Confidence Propagation Neural Network (BCPNN) Bate et al. (1998) is a Bayesian neural network inspired by Bayes' theorem, which uses probabilistic inference for ADR signal detection. Sequential Probability Ratio Test (SPRT), Evans et al. (2001), is a hypothesis test and works by minimizing the average number of samples (like patient records) needed to attain a conclusion with a specific level of statistical certainty.

These standard traditional methods calculate the likelihood of a drug reaction occurring as a result of a drug being administered to a patient. These methods form the foundation for ADR prediction in the field of pharmacovigilance. With the advent and establishment of artificial intelligence and machine learning models, it evolved toward models making predictions of ADR signals with efficient results.

### 2.2 Deep learning models for ADR signal detection

Deep learning is a subset of machine learning, and the models are built on multiple layers of neural networks that mimic and act like a human brain to learn complex patterns from data. ADR signal prediction over time has evolved from traditional standard methods, which are based on statistics and probability, to deep learning models, which are built after training them on vast amounts of data.

Chung and Lin (2025) proposed a novel method called Adverse Drug Reaction-Deep Q-learning Positive-Unlabeled Learning (ADR-DQPU), which detects ADR signals using deep reinforcement Q-learning. Instead of directly using the FDA Adverse Event Reporting System (FAERS) Kass-Hout et al. (2016) dataset, the authors used the ADR dataset generated from the FAERS dataset because it had duplicate reports, noise, and false positives. Each ADR cube contains the drug's ATC code, ADR name, and four statistical counts (a, b, c, d) from a 2 × 2 contingency table. They also used SIDER (Side Effect Resource), Kuhn et al. (2016), and BioSNAP (Stanford Biomedical Network Dataset Collection), Zitnik et al. (2018) to make the model learn about drug-ADR relationships. They used a deep Q-Network (DQN) to detect ADR signals, which, given a drug-ADR record, flags it as 1 if it is an ADR signal and 0 if it is not an ADR signal. The environment checks if the decision is right and gives a reward to the agent, +1 for a correct decision and −1 for a wrong decision. The proposed model outperformed many traditional methods and had an overall accuracy of 95%, a precision of 91%, and an F1 score of 90%.

Yan et al. (2022) introduced a computational approach called Predicting Drug-Drug Interactions Based on Integrated Similarity and Semi-Supervised Learning (DDI-IS-SL) to predict DDI by constructing drug similarity matrices from various drug information databases and applying a Regularized Least Square (RLS) model. They built a high-dimensional binary feature vector for each drug, where each dimension indicated the presence or absence of a specific feature. Then they formed a feature-based drug similarity matrix and used cosine similarity to measure similarity between two drugs. Using the drug similarity matrix as the input, an RLS classifier was applied to predict whether a drug pair interacts. Relational initial scores were calculated through performing node-based drug network diffusion methods to calculate DDI for unknown drugs, too. They evaluated their model with the AUC (Area Under Curve) metric, where, by 5-fold cross-validation, AUC was 0.9691, and by 10-fold cross-validation, AUC was 0.9745. Also, their proposed method had the best prediction performance in the *de novo* drug validation, with an AUC of 0.9292.

Reps et al. (2014) put forward a framework that brings together a range of existing techniques, like Mining Unexpected Temporal Association Rules given the Antecedent (MUTARA), Unexpected Temporal Association Rules (UTAR), Negating Temporal Association Rules, and Temporal Pattern Discovery (TPD) with web-scraped data, metric learning, and semisupervised clustering. Their work mainly focused on predicting rare and serious ADRs caused by popular drugs. Their model predicts the ADR, which occurs immediately after the drug is administered to the patient, and they label this event by scraping information from the internet. These medical events receive labels through metric learning and semisupervised clustering. They then use a filter to remove those medical events that are not true ADR, and the final dataset comprises those events that show unexpected recurrence within a period of 30 days. Their proposed method achieved an average rank of 143.75, while the other methods, such as TPD, achieved an average rank of 344.69, highlighting UTAR's Negating TARs (HUNT) with an average rank of 791 and MUTARA with an average rank of 2,385.73.

ChandraUmakantham et al. (2024) proposed a deep learning method that leverages a Graph Neural Network (GNN) to investigate the spatial and physical properties of drugs through molecular graph representations. Here, individual atoms correspond to nodes, and the chemical bonds between them are encoded as edges. To utilize the dynamics of a chemical reaction and the spatial and physical features of both reactants, they developed a dual GNN hybrid model with a 2-stage training phase. They used the TwoSides Tatonetti et al. (2012) polypharmacy side effects dataset from the Therapeutic Data Commons (TDC) dataset. Their proposed model outperforms other ADR prediction models by achieving 90% accuracy and 75% precision on the test dataset. A case study on the DrugBank dataset was performed to validate their results, and achieved a result of 99.2% in accuracy, precision, and F1 Score.

Bang et al. (2021) proposed the Graph Feature Attention Network (GFAN) to predict the side effects of polypharmacy. Additionally, the researchers incorporated an attention mechanism that placed different levels of importance on selected target genes and drug features, allowing for easier interpretation of results from the GFAN. Another significant aspect of the GFAN is that the authors created a new graph using the line graph concept to move beyond standard drug-drug interaction networks to connect them in a way that would allow polypharmacy prediction, and thus, the GFAN was framed as a node-based classification problem. The model was evaluated on benchmark node classification datasets and achieved test accuracies of 90.68% on IRIS, 89.40% on DIGIT, and 95.5% on USPS, demonstrating competitive performance with standard Graph Attention Networks. In polypharmacy side-effect prediction experiments, GFAN was capable of carefully extracting target genes contributing to each predicted side effect, providing the key important factors for domain experts.

Luo et al. (2025) proposed the Atomic 3D Position Encoding and Elastic Message Passing Graph Neural Network model (A3DPE-EMPGNN), which addresses the limitations of existing GNN-based methods that ignore the 3D structures of atoms within drug molecules and the impact of noise in GNNs. The authors created a model that has an atomic features network that incorporates 3D position encoding, using the centroid position of a molecule as the basis for measurement. They developed a model that has a molecular feature network and incorporates multi-head attention that captures interaction information between pairs of drug molecules. They also incorporated an adversarial attack detection and defense strategy in order to enhance the strength of the proposed model and utilized both supervised and contrastive loss learning during the model optimization process. On the ZhangDDI dataset, the A3DPE-EMPGNN model attained an accuracy of 98.61%, an area under the curve (AUC) score of 98.85%, an average precision (AP) of 99.24%, and a F1 score of 99.16% is reported. For the ChCh-Miner dataset, an accuracy of 98.27%, an AUC of 98.62%, an AP of 98.96%, and an F1 score of 98.91% is reported.

Nguyen et al. (2024) proposed CentSmoothie, a central-smoothing Hypergraph Neural Network (HGNN) for predicting DDIs that addresses the limitations of traditional GNNs, which represent side effects as independent one-hot vectors without capturing their relationships. CentSmoothie improves on the typical discrete representation of each associated drug by learning a relative weight of the combination of properties among the two associated drug nodes. Each associated drug node was encoded as a binary representation vector of 2,329 dimensions with a total of 881 features representing their chemical substructure and 1,448 features indicating protein-drug interaction with a unique protein. Evaluation of CentSmoothie was performed using three actual DDI datasets (TWOSIDES, CADDDI, JADERDDI) to compare it with the established approaches of graph-based neural networks (MLNN, MRGNN, SpecConv, Decagon, HPNN). For the TWOSIDES dataset, CentSmoothie achieved an area under the curve (AUC) of 0.9348 and an area under the precision-recall Curve (AUPRC) of 0.8749. For the CADDDI dataset, the proposed model achieves an AUC of 0.9846, demonstrating an ability to distinguish between drug-drug interaction pairs and non-interactions. The model also reaches an AUPR of 0.8230, which is significant as it proves the framework remains precise even when identifying rare or infrequent side effects. For the JADERDDI dataset, CentSmoothie achieved an AUC of 0.9684 and AUPR of 0.6044. Thus, providing improved predictive accuracy over traditional approaches to modeling hypergraph neural networks and graph-based approaches, particularly for predicting rare side effects.

Sounack et al. (2025) proposed BioClinical ModernBERT, a comprehensive long-context encoder for biomedical and clinical NLP. The model was developed through continued pre-training of ModernBERT on the largest biomedical and clinical corpus to date, exceeding 53.5 billion tokens from diverse sources, including PubMed, PMC, and 20 clinical datasets from multiple institutions, domains, and geographic regions. Both base (150M parameters) and large (396M parameters) versions were released. The model supports context lengths up to 8,192 tokens and outperforms previous biomedical and clinical encoders across multiple downstream tasks, including classification and named entity recognition. While the current study employs Bio_ClinicalBERT, future iterations of our framework could benefit from adopting such long-context encoders for processing longer and more complex clinical narratives.

The benchmark datasets used in this research domain are discussed below. Tatonetti et al. (2012) established the OFFSIDES dataset, a collection of 438,801 off-label side effects for 1,332 drugs. It identifies high-confidence adverse reactions that were statistically significant but missing from official documentation. Tatonetti et al. (2012) extended their earlier OFFSIDES work by releasing the TWOSIDES dataset, which focuses on single-drug reactions to adverse effects that occur when two drugs are taken together simultaneously. The dataset covers 557 drugs and 964 side effects, covering 49,677 unique drug-drug pairs that collectively produce over 3.6 million drug-drug-side effect combinations. Its structure follows the same format as OFFSIDES, with the only distinction being that the drug identifier and drug name fields appear twice to accommodate both drugs in the interacting pair, while all other statistical features carry the same meaning and interpretation as in OFFSIDES. The features of this dataset are,

- *drug*_*rxnorm*_*id* – RxNORM identifier for the drug
- *drug*_*concept*_*name*- RxNORM name string
- *condition*_*meddra*_*id*- MedDRA identifier for the side effect
- *condition*_*concept*_*name*- MedDRA name string
- *A*- The number of reports in which the side effect was recorded for the drug
- *B*- The number of reports for the drug in which the side effect was not recorded
- *C*- The number of reports for other PSM-matched drugs in which the side effect was recorded
- *D*- The number of reports for other PSM-matched drugs and other side effects
- *PRR*- Proportional reporting ratio, calculated as $PRR\;=\;\frac{A/\left(A+B\right)}{C/\left(C+D\right)}$
- *PRR*_*error*- Error estimate of the PRR
- *mean*_*reporting*_*frequency*- Proportion of reports for the drug that report the side effect, given by *A*/(*A* + *B*)

The FDA Adverse Event Reporting System (FAERS) is another dataset, which is a widely used database in pharmacovigilance. It is maintained by the U.S. Food and Drug Administration (FDA) and gathers real-world reports of medication errors and adverse events that occur once a drug has been approved and released to the market. The FAERS database is organized into relational files. The attributes in this dataset include:

- DEMO (Demographics): Basic patient information and administrative data for each report. The features of this table are primaryid, caseid, age, age_unit, sex, weight, wt_unit, country_code, event_dt.
- DRUG (Drug Information): Details for all medications reported in a specific case. The features of this table are primaryid, drug_seq (sequence number), role_cod (Primary Suspect, Secondary Suspect, Concomitant, or Interacting), drugname, val_vbm (validated trade name or verbatim), route, dose_amt, dose_unit, dose_freq.
- REAC (Adverse Reactions): All adverse events associated with the report are coded using the Medical Dictionary for Regulatory Activities (MedDRA) Preferred Terms (PT). The features of this table are primaryid, preferred term for the reaction (pt), and drug_rec_act.

### 2.3 Clinical decision support system

CDSS is an important framework for improving management of ADRs through use of evidence-based information delivered at the time of care. The CDSS integrates automated detection of ADRs with the risk assessment process, thereby minimizing inefficiencies in the workflow associated with pharmacovigilance. The CDSS can generate actionable recommendations based upon the synthesis of patient-specific data, including dosage adjustments and discontinuance of drugs, leading to a decrease in potential treatment-related injury and improvement in patient safety.

Gowtham et al. (2025) proposed a multimodal AI healthcare assistant that identifies the symptoms of a patient and recommends a medical specialist using text, voice (audio), or prescription images as input. It is a retrieval-based specialist recommendation system, which uses Retrieval-Augmented Generation (RAG). The authors used a variant of Bio_ClinicalBERT to map user input to the medical terminology, using Named Entity Recognition (NER) and Tesseract OCR to extract the raw text. The final output is a suggestion of which type of doctor or specialist the patient needs to consult based on the medical records. The authors reported a precision of 0.96, a recall of 0.78, and an F1-score of 0.86.

Krayem et al. (2026) published a paper titled “Clinical Reliability and Concordance of Drug-Drug Interaction Tools as Clinical Decision Support Systems in Patients With Epilepsy.” They analyzed how well various drug-drug interaction tools (DrugBank, Edmonton, Alberta, Canada, Drugs.com, mediQ, Medscape, and WebMD) perform in helping healthcare providers identify potential drug interactions with respect to the prescribed medications used in combination with one another for patients diagnosed with epilepsy and receiving antiepileptic medications. Specifically, these interactions were evaluated for consistency and clinical appropriateness of the warning provided by the external drug interaction checkers for different patients, as reported. The researchers concluded that there was significant variability in the accuracy of DDI detection rates from the different tools; however, the rates of DDI detection were significantly low across all the tools, with DDI detection rates ranging from 51.4% to 84.3%, and severe DDI rates ranging from 2.1% to 17.8%. In addition, the overall rate of concordance between the five DDIs was very low at 6.4%.

Pirnejad et al. (2019) proposed a clinical context-based methodology for developing a potential drug-drug interaction (pDDI) CDSS tailored for kidney transplant recipients, aiming to reduce alert fatigue caused by clinically irrelevant notifications. The researchers collected medication data from 595 patients across 788 clinical visits and employed the Medscape multi-drug interaction checker to identify pDDIs. Among the 52 most frequent interactions, only 33 were confirmed as clinically relevant through nephrologist consultation and Stockley's Drug Interactions reference. Thirty-three context-sensitive alert system modules were developed and coded according to their category (contraindicated, serious, significant), along with an EHR system that was developed in-house by the authors of this article. A major feature of this system was its ability to be customized based on the physician's preference to enable or disable alerts at the physician's and patient's level, so that the physician could have the flexibility to customize and/or disable the alerts without disrupting the workflow of the physician. In addition, this system provided passive awareness of previously indicated interactions.

Lu et al. (2024) introduced the ClinicalRAG, which is a multi-agent system that combines retrieval-augmented generation with a heterogeneous mix of medical knowledge in order to support clinical decision-making. Their approach starts by pulling out key medical entities from the user's input, then retrieves relevant details from structured sources like knowledge graphs and medical databases, as well as unstructured sources. When evaluated on a subset of the Chinese Biomedical Language Understanding Evaluation (CBLUE) Electronic Health Records (EHR) dataset, ClinicalRAG improved diagnostic accuracy in several LLMs. For instance, GPT-4.0 (an OpenAI model) accuracy increased from 82.78% to 84.94%, and GPT-3.5-Turbo (another one of OpenAI's models) went up from 80.04% to 81.75%. The improvement in accuracy shown by both models demonstrates the importance of retrieval-based methods in strengthening clinical applications.

## 3 Methodology

In this work, we propose a multi-stage learning framework called DrugPred, which includes a baseline ADR association learning model, a graph-based Drug-Drug Interaction modeling module, a transformer-based ADR risk prediction component, and a recommendation module for clinical guidance. We trained and tested our model on different datasets, such as OFFSIDES by Tatonetti et al. (2012), which has features to predict ADR reactions for a single drug, and TwoSIDES by Tatonetti et al. (2012), which is used to predict ADR reactions when multiple drugs are administered to a patient. The baseline ADR association model is used to find drug-ADR association probabilities. These learned associations are incorporated in a Graph Neural Network (GNN) to capture multiple drug interaction-induced ADR signals.

**Figure 1:**
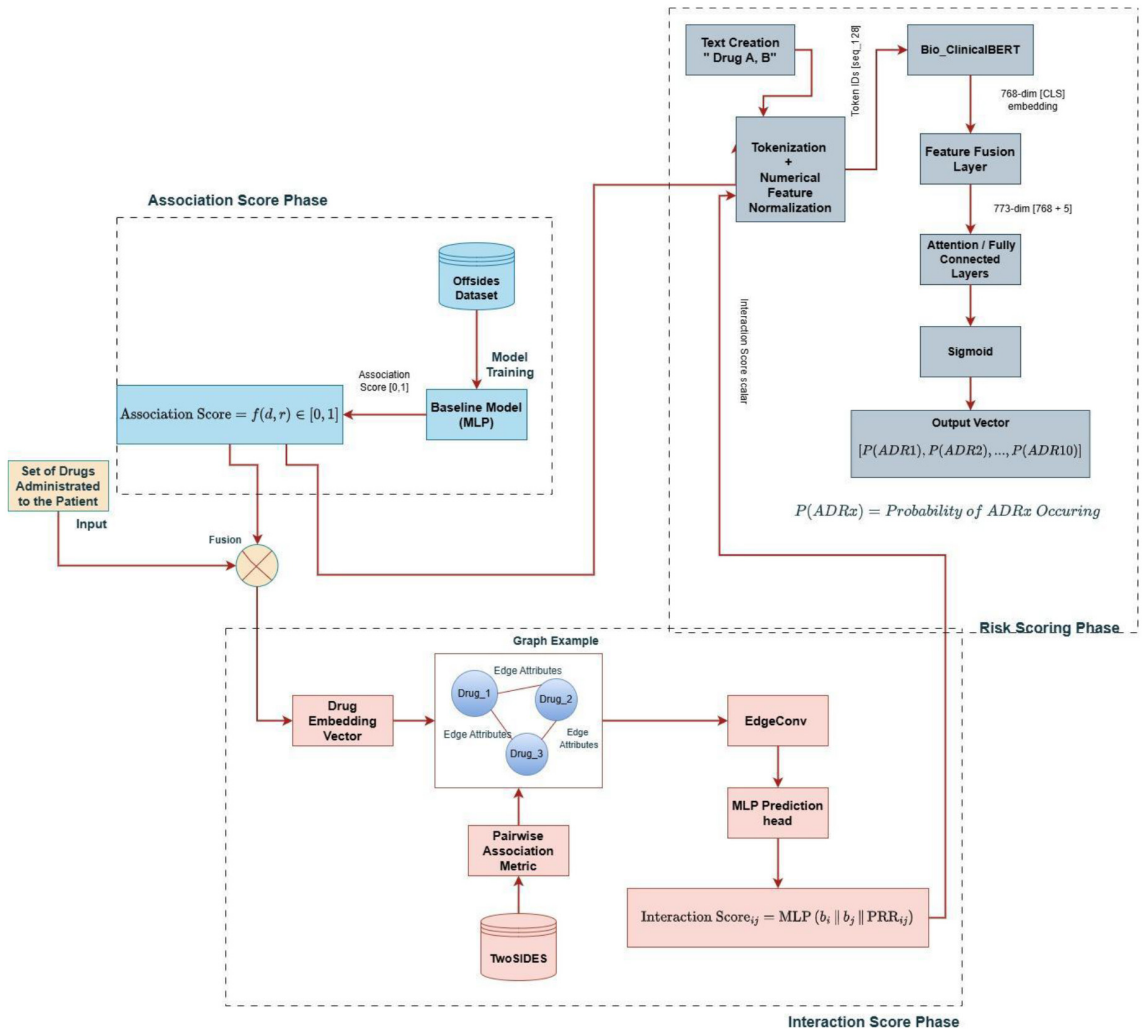
Proposed DrugPred architecture—the proposed four-phase pipeline begins with Phase 1 (Baseline MLP), which computes single-drug ADR association scores from OFFSIDES. These scores are passed to Phase 2 (EdgeConv-GNN), which models pairwise drug-drug interaction signals from TwoSIDES to produce interaction scores. Phase 3 (Bio_ClinicalBERT) fuses a clinical text embedding with numerical features from Phases 1 and 2 to produce a risk score in [0, 1] per drug-pair ADR combination.

The proposed framework is shown in Figure 1. This figure provides an overview of how we define our DrugPred framework to produce medication associations based on patient-reported drug-ADRs through patient medical history, and extract a complete list of medications that each patient has been prescribed (*P* = {*d*1, *d*2, ..., *dn*}) and their respective total number of drugs prescribed to each patient, and map from these medications (*D*) to unique drug-drug-ADR (D-D ADR) triplet forms.

In Phase 1, the Baseline MLP is trained on contingency table-derived features from the OFFSIDES dataset, specifically A, B, C, D, mean_reporting_frequency, total_counts, A_over_total, A_over_B, A_over_C, and BC_over_A. Binary labels are assigned using the threshold PRR ≥ 2.0 AND A ≥ 3, consistent with standard pharmacovigilance signal detection criteria; PRR itself is not passed as an input feature. The Association Score phase is used to find the association score, which is the probability of ADR for a particular single drug. We extract and generate the usage statistics (separate Proportional Reporting Ratio (*PRR*) value, *log*_10_(*PRR*) value, and co-occurrence value) from all the medications that were in a patient's medical record using the OFFSIDES dataset, which allows us to develop drug-ADR associations within our MLP based on the PRR function (*f*(*d, r*) ϵ [0, 1]) that provides us with an independent chance of experiencing ADRs from taking an individual prescription drug alone as well as generating a linear regression between the input (prescription drug) and the output (ADR) to develop our baseline association score *f*(*d, a*). The association scores for each drug-ADR pair is fused with the medical records of a patient in the feature fusion.

This forms a Drug embedding vector, which, along with the Pairwise Association metric derived for each drug from TwoSIDES Tatonetti et al. (2012) is the input to form the Drug-Drug Interaction Graph, as shown in the interaction score phase. In Phase 2, the EdgeConv-GNN operates on node features derived from the Phase 1 association scores (association_score_clipped) and edge features comprising the drug-pair prior scores, their maximum and absolute difference, a squashed transformation of log(PRR) as tanh(log_PRR/2), a normalized co-occurrence count log(1+pair_A_k)/5, and a capped co-occurrence frequency, which is seven features in total. The transformed PRR term serves purely as a relational edge attribute and is not used for labeling. To account for the more complex pharmacodynamic interaction that may result from a patient being placed on multiple medications, i.e., polypharmacy, at the same time, the DrugPred Framework is building medication-specific drug-ADR associations from the OFFSIDES dataset. We use the TwoSIDES dataset to construct a drug interaction graph (*G* = (*V, E*)) that contains either a patient-specific directed edge or an undirected edge representing each pair of medications. All edges will have a rich array of patient-ADR co-occurrence-related pharmacovigilance statistics associated with them. An EdgeConv-based Graph Neural Network (GNN) is trained on this graph, learning topological interaction embeddings through iterative message passing across drug neighborhoods and producing a relational interaction score (*MLP*([*b*_*i*_ || *b*_*j*_ || *PRR*_*ij*_]) for each drug-drug-ADR triple that captures the amplified ADR risk arising from drug co-administration. Interaction scores are the predicted probability that the drug pair is associated with an ADR risk.

This forms the Drug interaction embeddings, which are the input to the Risk score modeling phase, where a transformer is used to predict risk scores for 10 ADRs chosen from the GNN. In Phase 3, the Bio_ClinicalBERT model receives a structured text input of the form “Drug A: {name}. Drug B: {name}. Observed ADR: {term}.” alongside five numerical features: association_score_A, association_score_B, interaction_score, pair_count, and co_occurrence_freq. The 768-dimensional [CLS] embedding from Bio_ClinicalBERT is concatenated with these five features to form a 773-dimensional fused representation, which is passed through an attention gate and a fully connected classifier. Raw PRR values and contingency table variables are entirely excluded from Phase 3 inputs. This gives the final output of this research paper, the ADR prediction probabilities for the patient based on a given set of drugs. The numerical pharmacovigilance signals from the previous phase are then integrated with high-dimensional textual representations generated by Bio_ClinicalBERT, which encodes the specific drug-ADR triplets as input text into rich contextual clinical embeddings. The attention gate combines different numerical and textual documentation used in pharmacovigilance and provides data-driven guidance on how to evaluate the risk of new adverse effects after medication has been prescribed based on clinical documentation from multiple sources. The Attention Gate mechanism acts as a learned filter that evaluates the fused representation of numerical pharmacovigilance scores and clinical text embeddings. It assigns larger weight to dimensions where there is strong statistical evidence of association with an adverse drug response, such as those that have elevated *PRR* values or high interaction values, and, conversely, downweights those dimensions that do not contribute significantly to discrimination. In effect, the gate learns to distinguish between feature combinations that are statistically significant indicators of an ADR and those that are background noise, ensuring that only the most clinically meaningful signals influence the final risk prediction.

Evidence is weighted by the mode of action for each ADR class instead of using a single weighting to determine whether a feature value contributes equally to compute the final prediction. Predicted ADR values represent the risk associated with 10 specific ADR classes for each potentially high-risk ADR identified in terms of probability (from zero to one). Predicted ADR probabilities are used to estimate the risk of developing an adverse event caused by the drug.

All predicted ADRs will be processed by a Clinical Decision Support System (CDSS) module that identifies the SOC where the ADR will most likely occur when a physician refers a patient to an appropriate specialist. To ensure that all referenced evidence is based on evidence-based clinical practice in the medical community, the CDSS searches its own pre-built database of publicly available sources that contain drug product labels from OpenFDA and published scientific literature from PubMed to retrieve the drug-ADR predictions in real-time. Using the evidence it retrieves from the CDSS, it will provide physicians with written recommendations regarding which specialists to refer patients for possible ADRs based on evidence-based decision-making.

We used the following methodology when designing the training dataset: 70% was allocated for training, 10% was allocated for validating the model's accuracy, and 20% was allocated for testing the accuracy of the model to predict ADRs. All samples are randomly distributed across the 10 ADR categories and stratified across all three data samples to maintain a consistent representation of classes.

The procedure for dataset preparation and model execution is shown in Algorithm 1. All hyperparameters, including optimizer type, learning rate, batch size, dropout rates, and number of epochs for the ADR Risk Score prediction phase, are detailed in Table 1 for reproducibility.

**Algorithm 1:**
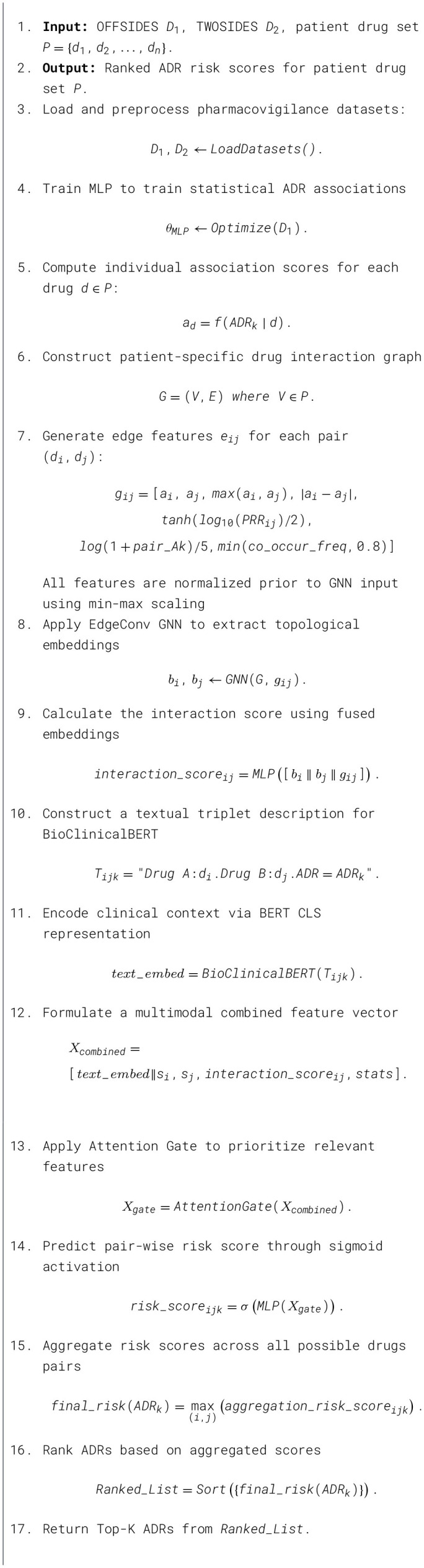
DrugPred Model

**Table 1:** Training configuration and hyperparameter settings.

Parameter	Configuration value
Loss function	Binary Cross-Entropy (BCE) loss
Optimizer	AdamW
Learning rate	2 × 10^−5^
Batch size	16
LR scheduler	OneCycleLR
Total epochs	5 (With early stopping)
Regularization	Dropout & gradient clipping

### 3.1 Input processing

The first stage of the treatment pipeline is focused on the collection and improvement of data related to patients. The main data stream of a patient's medical record serves as an entry point for the particular test scenarios to be reviewed by this system in order to identify potential ADRs. The cleaned data will serve as a basis for future instances of fusing and modeling activities through graph-based methods. These risk scores are later fed into the clinical decision support module in Phase 4, where high-risk ADRs are mapped to System Organ Classes, and corresponding specialist referrals are generated with evidence retrieved from OpenFDA and PubMed, ensuring that the pipeline delivers not just a prediction, but an actionable clinical recommendation grounded in real-world drug safety literature.

### 3.2 Baseline ADR association learning

**Figure 2:**
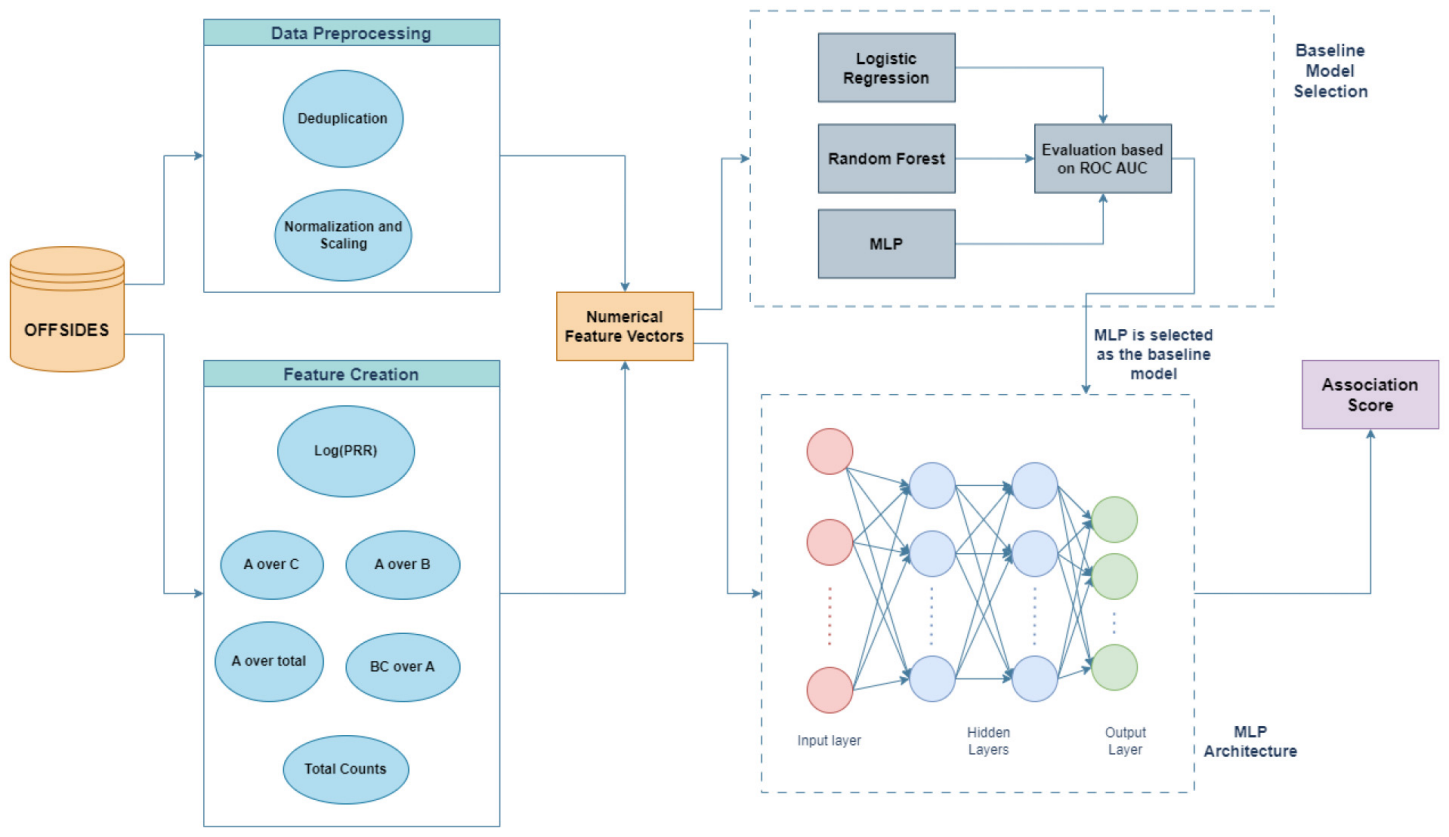
Workflow of ADR association learning model—baseline ADR association learning. Raw OFFSIDES drug-ADR records are deduplicated and preprocessed through normalization and feature engineering to produce 10 input features, including contingency table values A, B, C, D, and derived ratios. Three baseline classifiers: Logistic Regression, Random Forest, and MLP are trained and evaluated on ROC-AUC, with MLP selected as the best performing model. The MLP outputs an association score *fθ*(*d, r*) ϵ [0,1] per drug-ADR pair, which is clipped to [ϵ, 1–ϵ] for stability and passed to Phase 2 as the single-drug ADR prior.

In this stage, we process raw OFFSIDES data to create drug-ADR association priors using statistical features and baseline ML classifiers, as shown in Figure 2. The pipeline generates association scores for ranking ADRs per drug, which enables us to monitor tasks like polypharmacy risk management for the respective drug. The OFFSIDES dataset undergoes data preprocessing and feature creation before it is evaluated with different baseline models.

To maximize training efficiency, the OFFSIDES dataset was deduplicated to retain only unique drug-ADR pairs for downstream feature extraction. Normalization and scaling are another data preprocessing step done here, where some of the values in the dataset are normalized and scaled up or down accordingly. This is essential because the features in the OFFSIDES dataset, like *A, B, C, D* (the four cells in the contingency table) and *PRR* can range from 0 to tens of thousands. If these values are fed into a Machine Learning model directly, the model will be heavily biased toward these large-scale features.

Parallelly, the feature creation process is done on the OFFSIDES dataset, where the following features are created and appended to the OFFSIDES dataset:

- *Log*_10_(*PRR*), which gives a relative reporting rate of ADR for the drug. We used the Logarithmic Function of *PRR* because the logarithmic function stabilizes variance and makes the distribution symmetric.
- *A* /*C*, which gives a ratio of drug-specific ADR count over background ADR count, and when this value is high, ADR is more concentrated in this drug, and if it is low, ADR is common everywhere.
- *A*/*B*, which gives within-drug ADR intensity, and if this value is high, ADR is frequent among this drug's reports, and if the value is low, ADR is rare for this drug.
- *A*/*Total*, which gives the global proportion of all reports attributable to this drug-ADR pair.
- (B^*^C)/*A*, which captures the instability factor.

After the mentioned preprocessing steps and feature creation steps were complete, we trained the modified OFFSIDES dataset with different baseline models: Logistic Regression, Random Forest, and MLP. We evaluated the models based on the ROC AUC metric and found that MLP performs the best among the tested baseline models. Therefore, we used MLP to create the association score for each drug-side effect pair, which is used in the next phase. Equation 1 describes the association score as follows:


$$\begin{array}{l} Association\;Score\;=\;f_{\theta }\left(d,r\right)\;\epsilon \;\left[0,1\right] \end{array}$$ (1)

where *f*_θ_ denotes the MLP classifier, *d* denotes a drug and *r* denotes an (ADR).

To avoid extreme probabilities and stabilize further modeling, the raw association score is clipped as shown in Equation 2:


$$\begin{array}{l} a_{clipped}\left(d,r\right)\;=\;min\left(max\left(a\left(d,r\right),\epsilon \right),1-\epsilon \right) \end{array}$$ (2)

where ϵ is a small constant.

### 3.3 Drug-drug interaction (DDI) modeling

**Figure 3:**
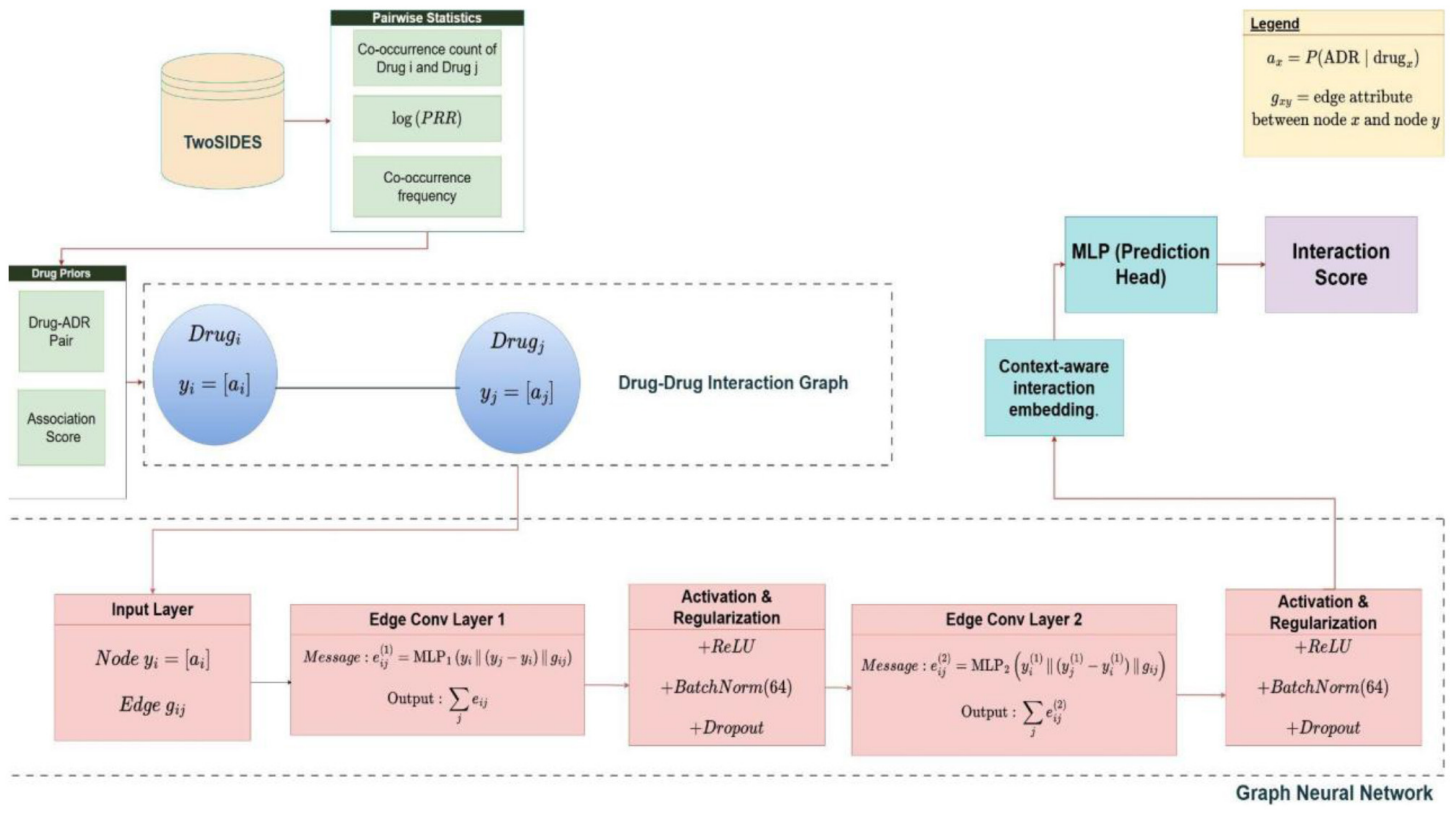
Drug-drug interaction modeling architecture diagram—each drug node is initialized with a 1-dimensional association score from Phase 1. Edges encode a 7-dimensional feature vector comprising pairwise prior scores, a squashed log-PRR term, normalized co-occurrence count, and capped co-occurrence frequency. Two EdgeConv layers perform message passing to produce 128-dimensional interaction-aware embeddings per drug node, which are concatenated with PRR and passed through an MLP head to produce an interaction score in [0, 1].

The DDI Modeling process is shown in Figure 3, where a Graph Neural Network (GNN) is utilized to identify ADRs when a patient is administered multiple drugs. The TwoSIDES Tatonetti et al. (2012) dataset is used here, which is the dataset that is built with data containing multiple drugs that affect various drug reactions rather than just a single drug exposure. Drug priors, which consist of Drug-ADR pairs and association scores (derived from Baseline ADR Association Learning) for each pair, are taken from the previous baseline modeling, and they are given as input in building the graph for Graph Neural Network (GNN). Pairwise statistics, which consist of co-occurrence counts of 2 Drugs, *log*_10_(*PRR*), and co-occurrence frequency are derived for every unordered drug pair (*i, j*) associated with a specific ADR.

A drug-drug interaction graph is constructed, where each node represents a drug and each edge corresponds to a potential interaction signal between a drug pair. For each unordered pair (*i, j*), pairwise prior features (*g*_*ij*_) can be computed by Equation 3,


$$\begin{array}{l} g_{ij}\;=\left[a_{i},a_{j},max\left(a_{i}a_{j}\right),|a_{i}-a_{j}|\right] \end{array}$$ (3)

where *a*_*i*_ = *P*(*ADR* | *drug*_*i*_) is the association score. Edges are connected between drug nodes only when the corresponding interaction statistic exceeds a particular threshold, which ensures the graph focuses on meaningful clinical interactions. The resulting graph is fed into a GNN-based EdgeConv architecture (Wang et al., 2019), which learns the node representation and interaction patterns. The EdgeConv architecture consists of the following parts:

- Input Layer, where the input, the Graph consisting of nodes and edges, is fed into this layer. The Node attribute is *y*_*i*_ = [*a*_*i*_] and the Edge attribute is *g*_*ij*_. This is fed into the GNN.
- Edge Conv Layer 1 is the first learnable layer, and message passing is done here. The message, which is information one node sends to another, is constructed as given in Equation 4.

$$\begin{array}{l} e_{ij}^{\left(1\right)}\;=\;MLP_{1}\left(y_{i}\;\|\;\left(y_{j}\;-\;y\right)\;\|\;g_{ij}\right) \end{array}$$ (4)

where *y*_*i*_ is drug *i*′*s* own prior, *y*_*j*_ − *y*_*i*_ is Risk contrast, *g*_*ij*_ is pairwise statistical evidence.

- Then, aggregation is done, where for each drug *i*, *Y*_*i*_ is calculated as given in Equation 5.

$$\begin{array}{l} Y_{i}\;=\underset{j\epsilon N\left(i\right)}{\sum }e_{ij}^{\left(1\right)} \end{array}$$ (5)

This combines all neighboring messages. The effect of neighboring drugs on the drug's ADR risk is learned in this layer.

- Activation and Regularization Layer is used to regularize the learning in the neural network. Here, the ReLU activation function is implemented to apply non-linearity and to help allow the model to understand complex interactions. BatchNorm is used to normalize feature distribution. Dropout is used to randomly drop some neurons or set them to zero, so that overfitting is avoided.
- Edge Conv Layer 2 is the second learnable layer, similar to the first Edge Conv layer, is used to construct messages and aggregate the messages. But in this layer, the message is constructed based on the learned features from the previous layer. The message *m*_*ij*_ is constructed as follows in Equation 6:

$$\begin{array}{l} e_{ij}^{\left(2\right)}\;=\;MLP_{2}\left(y_{i}^{\left(1\right)}\;\|\;\left(y_{j}^{\left(1\right)}-\;y_{i}^{\left(1\right)}\right)\;\|\;g_{ij}\right) \end{array}$$ (6)

where $y_{i}^{\left(1\right)}$ is drug *i*′*s* own prior after EdgeConv Layer 1, *y*_*j*_ − *y*_*i*_ is Risk contrast after EdgeConv Layer 1, *g*_*ij*_ is pairwise statistical evidence.

- Then, aggregation, similar to the first layer, is done, where for each drug *i*, $Y_{i}^{\left(2\right)}$ is calculated as given in Equation 7.

$$\begin{array}{l} {Y_{i}}^{\left(2\right)}\;=\sum_{j\epsilon N\left(i\right)}e_{ij}^{\left(2\right)} \end{array}$$ (7)

- Finally, $Y_{i}^{\left(2\right)}$ is passed onto an activation & regularization layer again, and the final output from the GNN is context-aware interaction embeddings.

For each drug pair (*i, j*), the learned interaction-aware embeddings, *b*_*i*_ and *b*_*j*_ are concatenated with the Proportional Reporting Ratio and passed through an MLP prediction head to estimate the interaction score as given in Equation 8.


$$\begin{array}{l} Interaction\;Score_{ij}=\;MLP\left(\left[b_{i}\;\|\;b_{j}\;\|\;PRR_{ij}\right]\right) \end{array}$$ (8)

This interaction score represents the predicted probability that the drug pair associated with ADR risk is caused by the drug-drug interaction rather than an independent drug.

The Phase 3 Bio_ClinicalBERT model was trained on a stratified sample of 50,000 records derived from the processed TWOSIDES dataset. We first constructed a processed dataset of 2,388,960 records from TWOSIDES, where each record corresponds to a unique (drug_A, drug_B, ADR_term) triplet. Each triplet was enriched with the following features: association_score_A and association_score_B (from the Phase 1 Baseline MLP), interaction_score (from the Phase 2 EdgeConv-GNN), as well as pair_PRR, pair_count, and co_occurrence_freq (directly from TWOSIDES).

Phase 1 association scores were joined to the TWOSIDES records by matching drug names. For drug pairs in TWOSIDES without a matching entry in OFFSIDES, a default association score of 0.0 was assigned. A stratified sampling strategy was employed to select the final 50,000 records, maintaining the original class distribution of approximately 64% negative and 36% positive labels. The resulting dataset was then split into training, validation, and test sets using a 72%/8%/20% ratio, with stratification by label.

### 3.4 ADR risk score prediction model

**Figure 4:**
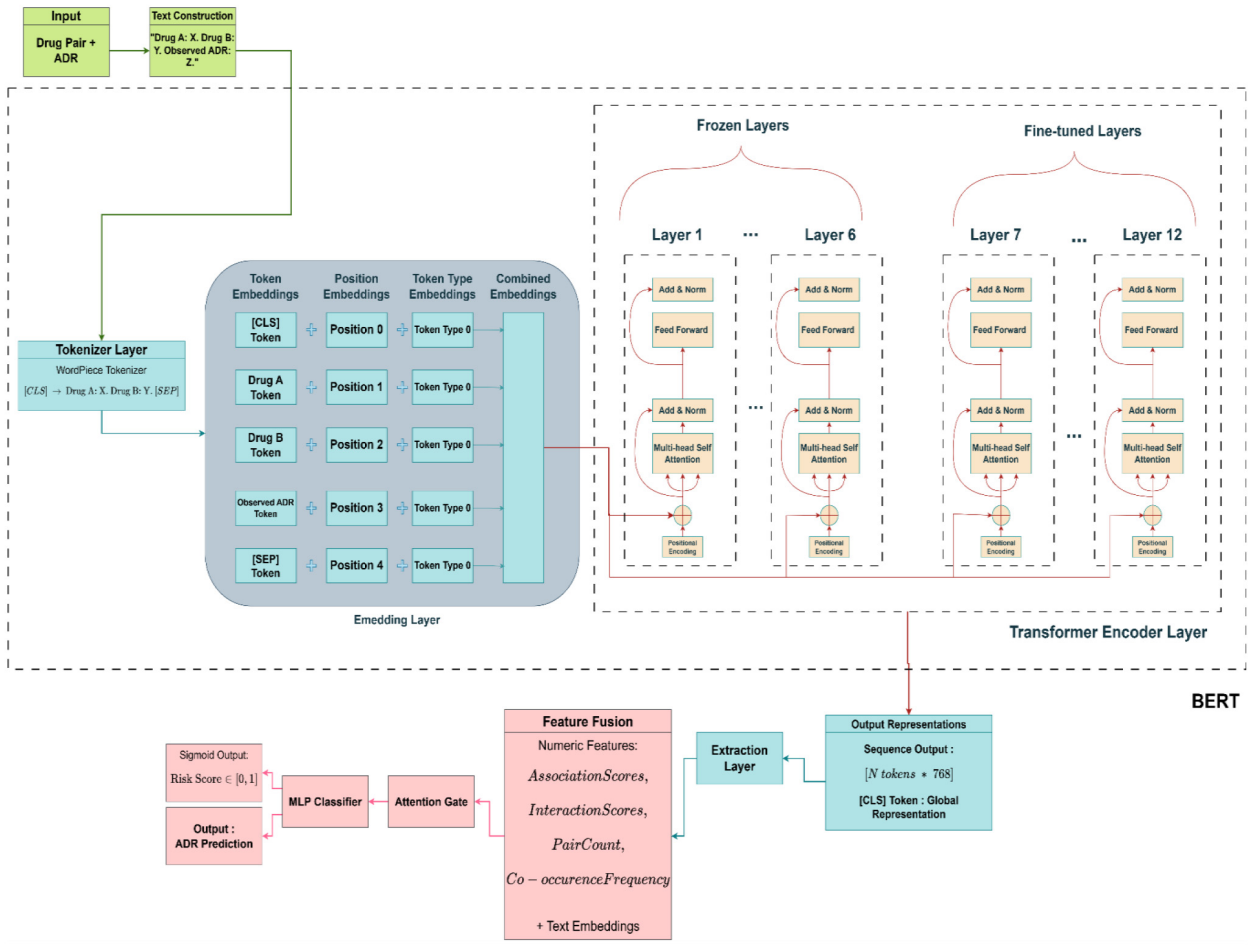
Architecture of ADR risk score prediction model—the Bio_ClinicalBERT encoder produces a 768-dimensional [CLS] embedding, which is concatenated with 5 numerical features to form a 773-dimensional fused vector passed through the AttentionGate and MLP classifier.

The final stage of the proposed framework, as shown in Figure 4, involves the final prediction of the 10 ADRs we chose from the GNN. The output is represented as risk scores, which compute the probability of occurrence of ADRs for a given drug pair by integrating information from previous phases. The input to this phase is generated from drug-drug interaction modeling, which contains drug records along with their interaction scores and other statistical evidence.

Each record consists of drug pair information (drug_A, drug_B), ADR term, association scores obtained from baseline modeling, interaction score from the EdgeConv Wang et al. (2019), and additional pairwise statistics such as co-occurrence count and frequency. This input is converted to a textual structured representation, which is in the format: “*Drug A*:*X*. *Drug B*:*Y*. *Observed ADR*:*Z*.″ The next step is tokenization, where the sentence is split into subword tokens. For example, a subword token looks like “[CLS] Drug A: Para ##ceta ##mol. Drug B: Ibu ##pro ##fen. ADR: Liver Toxicity. [SEP],” where [CLS] is a special classification token, [SEP] denotes the end of a sequence, and unknown words are broken into subwords.

The tokens are passed to the embedding layer, where three types of embedding take place: Token Embedding, which represents the semantic meaning of each token, Position Embeddings: encode the position of tokens in the sequence, and Token Type Embeddings: indicate sentence segments (all zeros in this case since single-sequence input is used). These embeddings are combined, and the resulting embedding vector has a dimension of 768 for each token. The combined embeddings are passed through a normalization layer to stabilize training and improve generalization.

The normalized embeddings are processed through a stack of 12 transformer encoder layers. The first 6 layers are frozen to retain the clinical knowledge, and the last 6 layers are fine-tuned accordingly to have task-specific learning. Each layer consists of the following:

- Multi-Head Self-Attention: This is when each token attends to all tokens in the sequence. Multiple attention heads learn different relationships. Mathematically, it's represented as shown in Equation 9:

$$\begin{array}{l} Attention\left(Q,\;K,\;V\right)\;=\;softmax\left(QK^{T}\;/\;\sqrt{d}\right)\;V \end{array}$$ (9)

where, *Q* is Query, which represents what the token is looking for, *K* is Key, which represents what features each token contains, *V* is Value, which represents the actual information passed forward, *d* is a scaling factor. Using this, context-aware representations are produced for each token.

- Add & Layer Normalization: This helps in stable gradient flow and avoids vanishing gradients.
- Feed Forward Network (FFN): Each token is passed through a fully connected network. It processes each token independently after attention has incorporated contextual information.

After passing through all the encoder layers, each token has a contextual embedding of size 768 and the output shape is sequence length^*^768. In the extraction layer, the embedding corresponding to the [CLS] token is extracted, which is $h_{cls}\;\epsilon R^{768}$. This vector represents the entire input sequence, capturing Drug A information, Drug B information, ADR context, and interaction semantics.

Next, feature fusion takes place, where numerical features like association score from baseline association score modeling, interaction score from drug-drug interaction score modeling, pair count, and co-occurrence frequency are combined with the textual embeddings, and the resulting vector size is 773 dimension. This is passed into the attention gate, where the model learns the importance of each feature dimension. It is then passed to an MLP classifier, where non-linear interactions are learned. Finally, the sigmoid activation function is applied, which gives a risk score in the range of [0, 1].

The model utilizes Binary Cross-Entropy loss, where labels are derived from the Proportional Reporting Ratio (PRR). PRR is used exclusively for label construction and is not used directly as a predictive input feature in the model training phase. The binary labeling of ADRs has been done using a PRR >= 2.0 threshold and uses the PRR statistics as input features. This is considered a well-established part of pharmacovigilance signal detection. Not only does this support the use of PRR as one component of the signal detection process, but it also demonstrates that Noguchi et al. (2021) also used features based on PRR to help detect signals related to drug-drug interactions.

The contribution of each component across the pipeline can be reasoned from the progressive performance observed across phases. The Baseline MLP (Phase 1), trained on OFFSIDES-derived contingency table features, establishes the foundational single-drug association signal upon which both subsequent phases depend. The EdgeConv-GNN (Phase 2) builds on this by modeling pairwise drug relationships through graph-based message passing over TwoSIDES, achieving a test AUROC of 0.9943, suggesting that relational modeling between drug pairs is a strong contributor to discriminative performance. The Bio_ClinicalBERT model (Phase 3) fuses the 768-dimensional [CLS] embedding with five numerical scores from the prior phases into a 773-dimensional representation. The attention gate then selectively weights each feature dimension before passing it to the classifier, effectively arbitrating the contribution of each upstream component (the association scores, interaction scores, and contextual embeddings) toward the final prediction, achieving a test ROC-AUC of 0.9930 and PR-AUC of 0.9882. These signals are then integrated via the Attention Gate, which assigns dynamic weights to the contributions from each phase before producing the final risk prediction. There is no overlap between label generation criteria and predictive input features to avoid circular dependency. Given below is the list of input features along with how PRR is used individually across the three phases.

Table 2 gives the input features used across each phase of the proposed DrugPred framework. Table 2 is divided into three sections, namely, the Baseline MLP, EdgeConv-GNN, and the use of Bio_ClinicalBERT with the use of the Attention Gate mechanism as used in phase 3. In phase 1, we implement the stated PRR methodology and make sure that it is not passed as an input feature. The input features used are stated. In phase 2, we use PRR only as a relational weighting signal, and it does not contribute to the label generation. Here, the TwoSIDES is used with the node features as stated. In phase 3, we implement pair_PRRs, which are excluded from input features. The input features are as stated, with the dimensions being 768 [Clinical BERT] + 5 [input features] = 773. Table 3 summarizes the notation used across these three phases, listing each variable alongside its description.

**Table 2:** Input features used across each phase of the DrugPred pipeline.

Phase	Model	Input features	PRR role
Phase 1	Baseline MLP (OFFSIDES)	Contingency table values (A, B, C, D), mean_reporting_frequency, total_counts, A_over_total, A_over_B, A_over_C, BC_over_A	PRR ≥ 2.0 AND A ≥ 3 used solely for binary labeling; not passed as input feature
Phase 2	EdgeConv-GNN (TwoSIDES)	Node features: association_score_clipped (per drug, per ADR) from Phase 1. Edge features: s_i_k, s_j_k, max(s_i, s_j), |s_i – s_j|, tanh(log_PRR/2), log1p(pair_A_k)/5, min(co_occur_freq, 0.8)	Log-transformed PRR is used only as a relational weighting signal in graph construction and does not contribute to label generation.
Phase 3	Bio_ClinicalBERT + Attention Gate	Text: “Drug A: {name}. Drug B: {name}. Observed ADR: {term}.” Numerical: association_score_A, association_score_B, interaction_score, pair_count, co_occurrence_freq. Fused dim: 768 + 5 = 773	Pair_PRR used only for labeling (PRR > 2.0); excluded entirely from input features

**Table 3:** Variables used and their description.

Variable	Description
*f*_θ_(*d, r*) ϵ [0, 1]	Association score between the drug *d* and adverse drug reaction *r*, predicted by the baseline MLP model
*a*_*clipped*_(*d, r*)	Clipped association score used to avoid extreme probability values
*g*_*ij*_	Edge feature vector between drug nodes *i* and *j* in the DDI graph
*y*_*i*_	Node feature vector (initial association score) of the drug *i*
*e*_*ij*_ *(1)*	The message passed from the drug *j* to drug *i* in the first EdgeConv layer
*e*_*ij*_ *(2)*	The message passed from the drug *j* to drug *i* in the second EdgeConv layer
$Y_{i}^{\left(1\right)}$	Aggregated node representation of the drug *i* after the first EdgeConv Layer
$Y_{i}^{\left(2\right)}$	Aggregated node representation of the drug *i* after the second EdgeConv layer
*b*_*i*_, *b*_*j*_	Context-aware embeddings of drug *i* and drug *j* after GNN
*Interaction Score*_*ij*_	Final interaction score between drug pair (*i, j*) predicted by the MLP head
*Risk Score*_*ijk*_	Predicted probability of ADR *k* occurring for the drug pair (*i, j*)

### 3.5 Specialist recommendation & clinical guidance

In this phase, the predicted ADR risk scores from the previous stage are transformed into clinical recommendations using a Retrieval-Augmented Generation (RAG) based framework. The objective is to match the ADRs to appropriate medical specialists and provide evidence-backed guidance for clinical decision-making. The input in this phase consists of drug pairs (Drug A, Drug B), predicted ADR, and risk scores from Phase 3. Each ADR is mapped to its corresponding System Organ of Class (SOC) using the MedDRA hierarchy. This mapping enables categorization of ADRs to an appropriate affected physiological system.

Each SOC is mapped to a relevant clinical specialist using a standard, medically accepted, predefined rule-based mapping. This ensures that each ADR is directed to the appropriate domain expert. Urgency level for each ADR is determined based on the predicted risk score and clinical severity of the SOC. The classification of the urgency levels is Immediate for high-risk and Life-Threatening SOC, Urgent for moderate to high risk, and Routine for lower risk. A patient-specific knowledge base is constructed dynamically using external clinical data sources such as FDA drug label data (through OpenFDA API) and PubMed abstracts (through Entrez API). Relevant sections, such as adverse reactions, drug interactions, warnings, and precautions, are extracted and segmented into smaller chunks for retrieval.

Each knowledge chunk is encoded using Bio_ClinicalBERT to generate dense vector representations. *chunk embeddings ϵR*^768^ are the embeddings with 768 dimensions, which are stored in a FAISS vector index for efficient similarity search.

For each ADR, a structured query is constructed, for example, “Drug combination: X and Y. ADR: Z. Clinical recommendation,” where the query is embedded and compared with stored vectors using cosine similarity. The *top* − *k* most relevant chunks are retrieved. The retrieved evidence is injected into a predefined template to generate structured recommendations. Each output includes the Specialist name, ADR, and Associated Risk score, Urgency level, Recommended clinical action, and Source citation. All tensor dimensions are explicitly annotated in figure captions.

## 4 Experimentation and results

In this section, we perform an experimental evaluation of the proposed DrugPred framework, which covers the datasets used, the experimental setup, and the results obtained across all stages of the pipeline. The performance of the framework is assessed using standard classification metrics and compared against baseline approaches to demonstrate its effectiveness in predicting ADR risks in multi-drug settings.

### 4.1 Experimental setup and dataset split

The proposed framework works with multiple medical datasets and a structured experimental setup to ensure reliable and generalizable performance in all stages. OFFSIDES Tatonetti et al. (2012) and the TwoSIDES Tatonetti et al. (2012) datasets were used for baseline ADR modeling and Drug-Drug Interaction (DDI) modeling, respectively. The data splitting strategy is as follows:

- Baseline Model: Random split and drug-held-out split to ensure model generalization to unseen drugs. A drug-held-out is a specific split where we test the model's ability to generalize to drugs it has not seen before. It is reserved specifically for the test phase.
- DDI Modeling: Model was trained with 7 ADRs, validated with 1 ADR (unseen), and tested with 2 ADRs (unseen).
- ADR Risk Prediction: The dataset was divided into 72% for training, 8% for validation, and 20% for testing. The training configuration is shown in Table 1.
- Objective Optimization: Binary Cross Entropy (BCE) Loss is implemented to handle multi-label risk classifications, thus allowing the output probabilities of independent adverse reactions to range between 0 and 1.
- Weight Optimization: AdamW is employed to handle decoupled weight decay. This way, all the parameters get a fair amount of regularization, ensuring that the structural parameters of the text embedding layers and the underlying graph network do not overfit to localized training distributions.
- Scheduling: A maximum learning rate of 2 × 10^−^5 is controlled via a OneCycleLR scheduler. This steps the step-size upward during initial phases to avoid the bad cases before slowly reducing the step-size to allow for proper convergence and prevent the model from avoiding the best cases during iterative message-passing.
- Convergence Controls: A combination of Dropout layers is used to prevent co-adaptation (interdependence of nodes) of features, Gradient Clipping to limit unexpected gradient spikes, and an Early Stopping monitor to halt optimization, the moment validation loss begins to diverge.

### 4.2 Baseline model evaluation

In the initial phase of experimentation, three baseline machine learning classifiers: random forest, logistic regression, and multi-layer perceptron (MLP) models were evaluated for their ability to learn drug-ADR associations from the OFFSIDES dataset.

The models were trained and tested under both random and drug-held-out splits. Logistic Regression achieved an AUPRC value of 0.935 and ROC_AUC value of 0.988, Random Forest achieved an AUPRC of 1.0 and ROC-AUC of 1.0, while the MLP reached an AUPRC of 0.999 and ROC_AUC of 0.999. These values are high and expected since the dataset, which was trained, is statistically perfect and structured. Even though Random Forest exceeded other models in terms of performance, MLP is chosen as the baseline model because Random Forest memorizes threshold-like patterns and overfits statistical artifacts, while MLP learns smooth probability surfaces and generalizes better.

The resulting scores were then clipped to ensure that extreme probabilities did not affect the GNN modeling. This phase successfully established the single-drug priors, providing the essential foundation for the relational analysis that is performed in the next phase.

### 4.3 Drug-drug interaction (DDI) performance

In the second phase of experimentation, a Graph Neural Network (GNN) based on the EdgeConv operator was implemented to model drug-drug interactions and estimate the risk of ADRs arising from drug combinations.

To ensure generalization in prediction, the model was trained on 7 ADRs, validated on 1 unseen ADR, and tested on 2 completely unseen ADRs. This setup evaluates the model's ability to transfer learned interaction patterns to new ADRs.

As shown in Table 4, on the validation set, it achieved an AUROC value of 0.9967, an AUPRC value of 0.9974, and an F1-score of 0.9653, indicating high discriminative capability and balanced precision-recall performance. On the test set, which consisted of unseen ADRs, the model maintained high performance with an AUROC value of 0.9943, AUPRC value of 0.9958, and F1-score of 0.9443. Training converged efficiently within 36 epochs using early stopping, with the best validation loss achieved at epoch 26.

**Table 4:** Interaction scoring phase results.

Dataset	Loss	Accuracy	F1-score	AUROC	AUPRC
Train	0.1567	0.9509	0.9484	0.9949	0.9962
Validation	0.1039	0.9664	0.9653	0.9967	0.9974
Test	0.1612	0.9472	0.9443	0.9943	0.9958

Further large-scale inference was performed to generate a comprehensive interaction score table, covering over 2.4 million (drug pair, ADR) combinations across 10 ADRs. The interaction scores showed a wide distribution with a mean of approximately 0.446 and a median of approximately 0.038, indicating the model's ability to differentiate between low-risk and high-risk interactions.

### 4.4 ADR risk score predictions results

In the final phase, the Bio_ClinicalBERT-based ADR Risk Prediction model is evaluated on its capability to determine the occurrence of ADRs for drug pairs. The model integrates the numerical features from previous phases and the semantic representations from the clinical text. We trained our model for 5 epochs, with continuous improvement observed over all the epochs. Only 5 epochs were set as the upper bound based on standard BERT fine-tuning techniques. With 110 million parameters being fine-tuned on 36,000 samples, additional epochs may cause overfitting, and generalization fails. The early stopping mechanism with patience = 2 ensures training terminates at the optimal epoch regardless of the 5-epoch ceiling. In practice, the model converges before reaching the maximum.

In Table 5, the loss decreases over epochs, denoting that the model is learning meaningful patterns without divergence. Validation F1 score increases over epochs, denoting better balance between precision and recall over time. Increase of precision over epochs denotes fewer false positives as training progresses, and the best model is obtained in Epoch 5.

**Table 5:** Risk scoring phase training and validation results.

Epoch	Train loss	Val accuracy	Val F1	Precision
1	0.4753	0.9173	0.8925	0.8368
2	0.1917	0.9330	0.9118	0.8651
3	0.1491	0.9497	0.9322	0.9045
4	0.1263	0.9570	0.9406	0.9341
5	0.1074	0.9620	0.9463	0.9523

**Figure 5:**
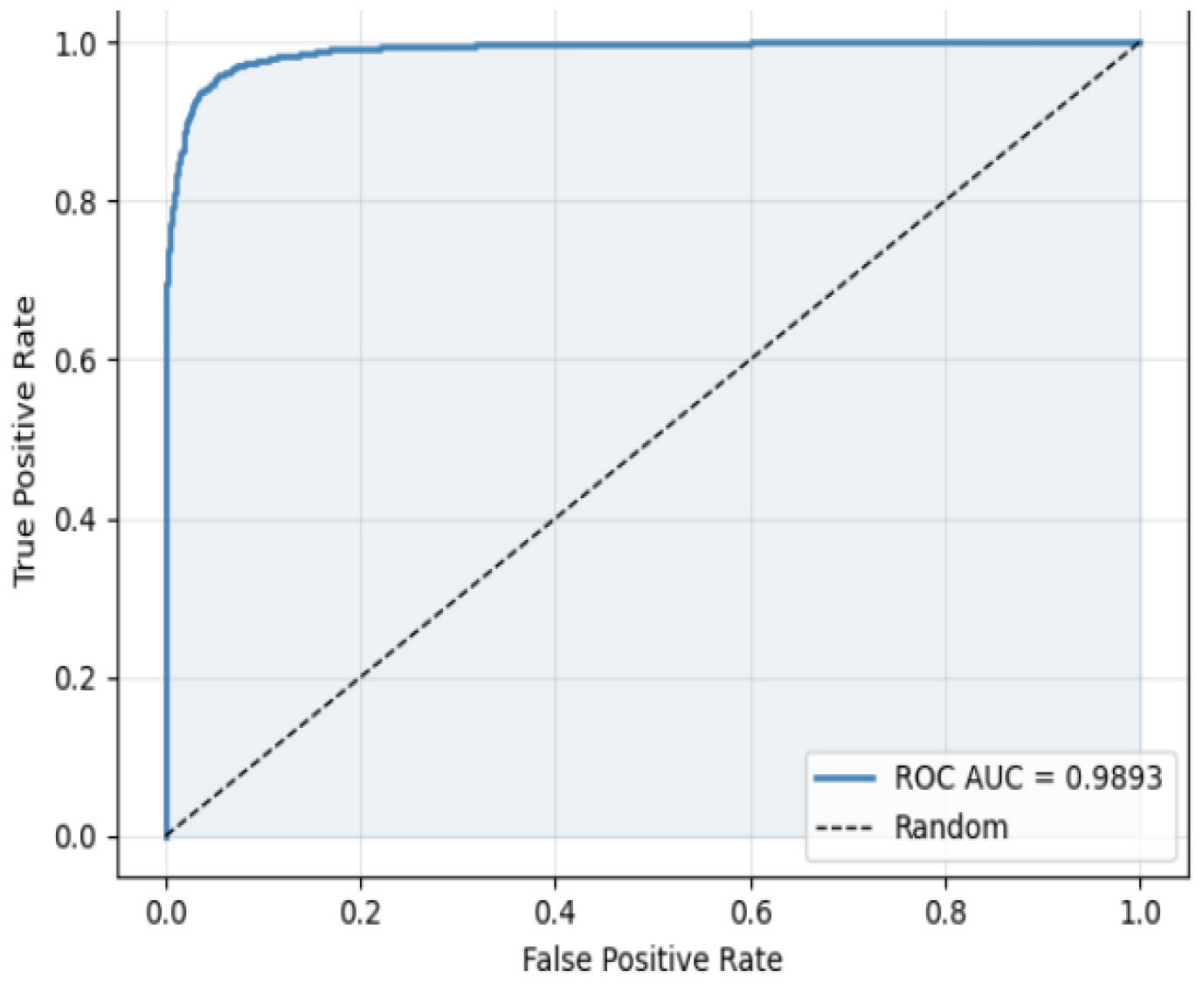
ROC curve of training results.

As shown in Figure 5, the ROC curve describes the ability of the model to discriminate between ADRs and non-ADRs at various classification thresholds. The x-axis of the curve plots TPR (True Positive Rate) against FPR (False Positive Rate) and therefore, the model would ideally classify all ADRs (true positives) and no ADRs (true negatives) in the upper-left corner of the plot.

The model achieves an ROC-AUC value of 0.9893, which suggests there is a 98.93% chance that the model will score an ADR higher than an equivalent non-ADR case (i.e., when selecting a sample from both classes at random, the ADR will score higher than the non-ADR). As indicated by the initial steep ascent of the model ROC curve toward the upper-left corner and low FPR values, the model successfully identifies most true ADR cases while returning very few false positives. The substantial differential or gap between the model curve and the random baseline supports the conclusion that the model produces accurate predictions at all the classification thresholds. The ROC-AUC value confirms that the Bio_ClinicalBERT model is reliable enough to classify an ADR as positive or negative based on text, indicating that using pharmacovigilance signals (such as structured clinical notes) embedded in clinical text will improve the prediction of an ADR from clinical text.

**Figure 6:**
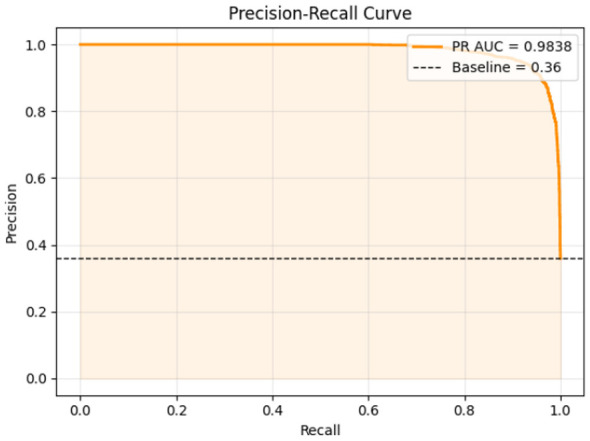
Precision-recall curve of training results.

As shown in Figure 6, the precision-recall curve evaluates how well our model performs with regard to the class imbalance that exists in our dataset (with 6,407 negative cases and 3,593 positive cases). The PR curve is more sensitive than the ROC curve to model performance on the minority positive class. Hence, the PR curve is more important than the ROC curve when it comes to detecting ADRs in clinical settings. The model achieves a PR-AUC of 0.9838, substantially above the baseline of 0.36, which represents the performance of a classifier that predicts ADR at the same rate as the class distribution. The curve maintains a precision of approximately 1.0 across the majority of the recall range, only declining sharply beyond a recall of approximately 0.85. When viewing the high precision area, we see that most of the time when a model predicts ADRs, it will do so correctly, and this high degree of precision is maintained as they are asked to detect an increasing number of true ADRs. Additionally, the wide gap between the model curve and the baseline reflects how well the model has the ability to predict the positive class, which supports the usefulness of combining the Bio_ClinicalBERT encoder and the attention gate to identify clinically significant relationships between drugs and ADRs.

**Figure 7:**
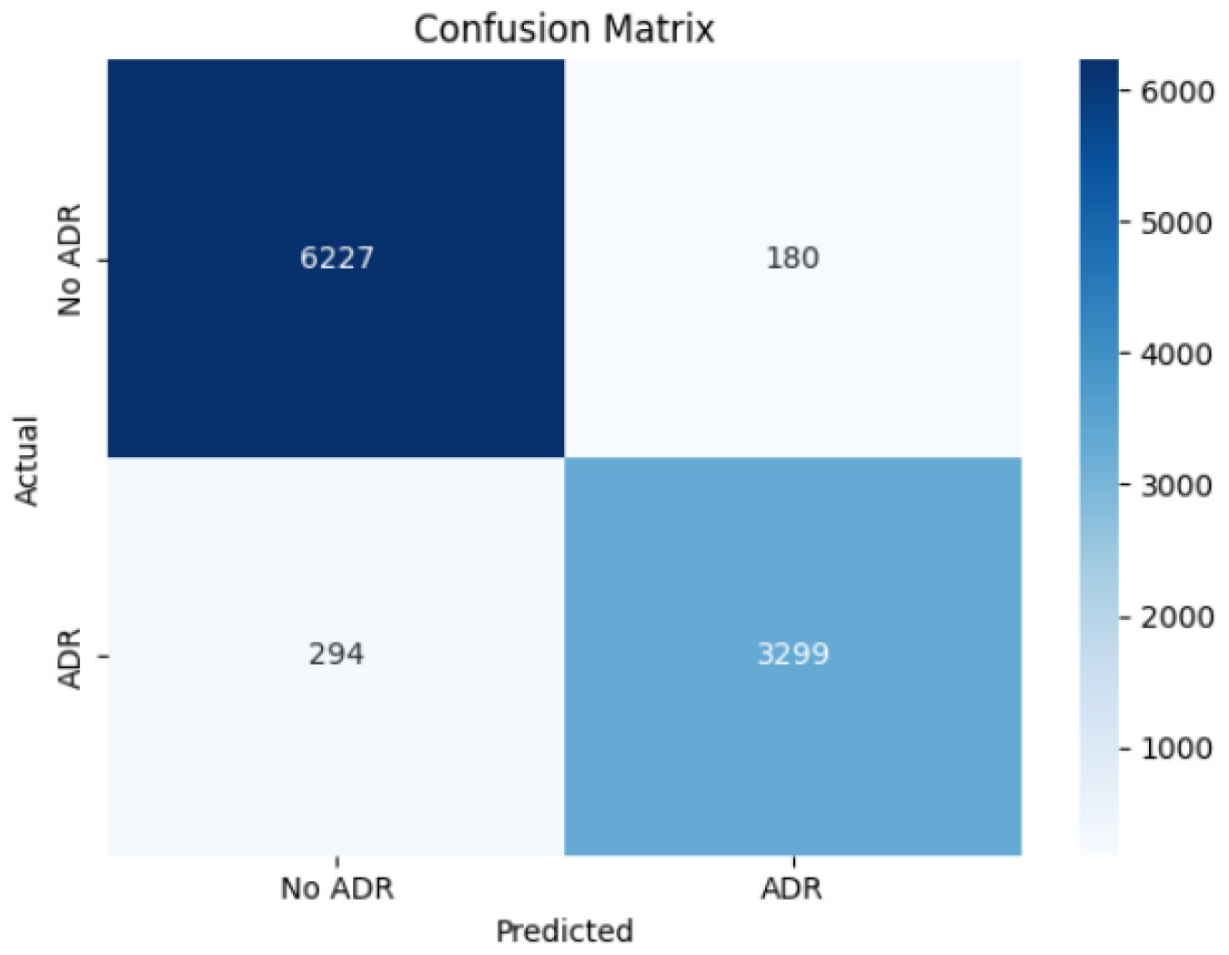
Confusion matrix for 10,000 samples.

From Figure 7, we see that both classes of results are classified well. In addition, the expected reliability of the model on the test set can be seen in that the model accurately classified 3,299 cases of ADR and 6,227 cases of No ADR. The model creates very few errors in classifying cases of ADR, with only 180 false positive alerts for ADR, which directly contributes to its good precision when determining whether an ADR has occurred. While there are 294 false negative classifications of ADR, the average false negatives are not high, which indicates that instead of producing at least one alert for every possible ADR, the model has leaned toward producing relatively accurate and confident classifications. Overall, these numbers demonstrate that this is an accurate model for use in the clinical setting and that to reduce the number of missed detections of ADR, the best way is to continue increasing the recall on the model's subsequent versions.

The model was trained and evaluated on a stratified sample (independent subgroups) of 50,000 records drawn from the complete TwoSIDES-derived interaction score dataset (2,388,960 rows), using a fixed random seed (random_state = 42) to ensure reproducibility. All sampling was performed prior to model training and evaluation. This sample was subsequently divided into training (36,000; 72%), validation (4,000; 8%), and test (10,000; 20%) subsets using stratified splitting to preserve the original class distribution uniformly across all three splits. The 10,000 samples reported in Figure 7 represent the complete held-out test set and are not a random subset or a *post-hoc* balanced evaluation sample.

Training was conducted for a maximum of 5 epochs with early stopping (patience = 2) based on validation F1-score. The best model checkpoint was selected at epoch 5. The validation set was strictly held out and generated via stratified splitting prior to any training.

As shown in Table 6, the accuracy is almost 96%, denoting that all the predictions are very efficient and reliable. F1 Score is 94%, which denotes a strong balance between precision and recall. ROC-AUC being 99% explains that our model has near-perfect class separability. Precision is 94%, which says the predictions have a low false alarm rate, and recall being 93% says most ADR cases are correctly detected. These results demonstrate that our proposed model achieves a high accuracy in distinguishing ADR vs. non-ADR cases and strong ranking capability as indicated by high ROC-AUC.

**Table 6:** Test set performance.

Metric	Value
Accuracy	0.9573
F1 score	0.9400
ROC-AUC	0.9930
Precision	0.9492
Recall	0.9310

Table 7 illustrates how consistently the model performs in both classes. The model has the same level of performance for both the “No ADR” and ADR classes, as they have a precision of 0.95 at a threshold of 0.5. The model achieves a recall of 0.97 for the “No ADR class” and 0.92 for the ADR class, indicating that it is slightly more sensitive to negative cases, which is consistent with the class distribution of 6,407 “No ADR” samples as against 3,593 ADR samples. The F1 scores of 0.96 and 0.93 for the two classes indicate that neither class has significantly traded off precision or recall, and the macro average and the weighted average scores of 0.95 suggest that the model does not have an inherent bias toward the majority class but treats both classes with the same degree of precision. Thus, the results also suggest that the model is a balanced classifier with good generalizability for both ADR outcomes.

**Table 7:** Classification report.

Class	Precision	Recall	F1-score	Support
No ADR(0)	0.95	0.97	0.96	6,407
ADR(1)	0.95	0.92	0.93	3,593
Accuracy	-	-	0.95	10,000
Macro accuracy	0.95	0.95	0.95	10,000
Weighted average	0.95	0.95	0.95	10,000

For this experiment, Phase 1 and Phase 2 were regenerated entirely without any PRR-derived statistics to evaluate model performance in the complete absence of PRR. The Phase 3 label in this ablation was constructed by thresholding the Phase 2 interaction_score, while interaction_score itself was withheld from the Phase 3 input features. Although the retained features (association scores, pair_count, co_occurrence_freq) do not include PRR, they are derived from the same underlying statistics used to compute interaction_score in Phase 2, and remain closely correlated with it. This correlation, rather than the absence of PRR, accounts for the near-perfect metrics shown in Table 8. These results should therefore not be interpreted as evidence of independent, PRR-free predictive performance.

**Table 8:** Ablation study: model training performance with and without PRR-derived features.

Metric	With PRR-derived features	Without PRR-derived features
Accuracy	0.9573	0.9966
F1-score	0.9400	0.9966
ROC-AUC	0.9930	1.000
PR-AUC	0.9838	1.000
Precision	0.9492	0.9990
Recall	0.9310	0.9942

Although the ablation study shows that the Bio_ClinicalBERT model maintains strong performance even without PRR-derived features (Table 8), PRR remains a crucial part of the DrugPred framework. We used PRR to create the ground truth labels for training across the phases of the pipeline. We considered a drug-ADR pair to be a positive signal only if its PRR was greater than 2.0 and it had at least three co-reported cases. This threshold is a standard practice followed in pharmacovigilance. PRR provides important clinical context by normalizing for background reporting noise and bias present in spontaneous reporting databases like FAERS. Even though the model can predict well without directly using PRR at inference time, it learned meaningful patterns from data originally labeled using PRR. Thus, PRR serves as the clinical foundation of our labeling strategy rather than just another input feature.

### 4.5 Specialist recommendation performance

In this phase, we used the final prediction results from the ADR Risk scoring phase, and we proposed a system that translates ADR risk scores into specialist recommendations using a Retrieval-Augmented Generation framework. We evaluated our recommendation system using a patient case involving Ethinyl Estradiol, Acetaminophen, and Epoetin Alfa drugs, and a total of 9 ADRs exceeding the risk threshold were identified and mapped to appropriate specialists based on System Organ Class (SOC). Out of these 9 ADRs, 4 ADRs were classified as IMMEDIATE, 4 as URGENT, and 1 as ROUTINE. The specialist routine was found to be clinically consistent. For example, Anemia was correctly matched to a Hematologist, and Dyspnoea to a Pulmonologist, both with immediate urgency level, and similarly Diarrhea and Vomiting were matched to a Gastroenterologist.

The retrieval component achieved an average cosine similarity of 0.921, which shows a strong match between the query and the knowledge base. Overall, the system showed the ability to generate accurate and prioritizable clinical interpretable recommendations, thereby providing a reliable decision support system.

**Figure 8:**
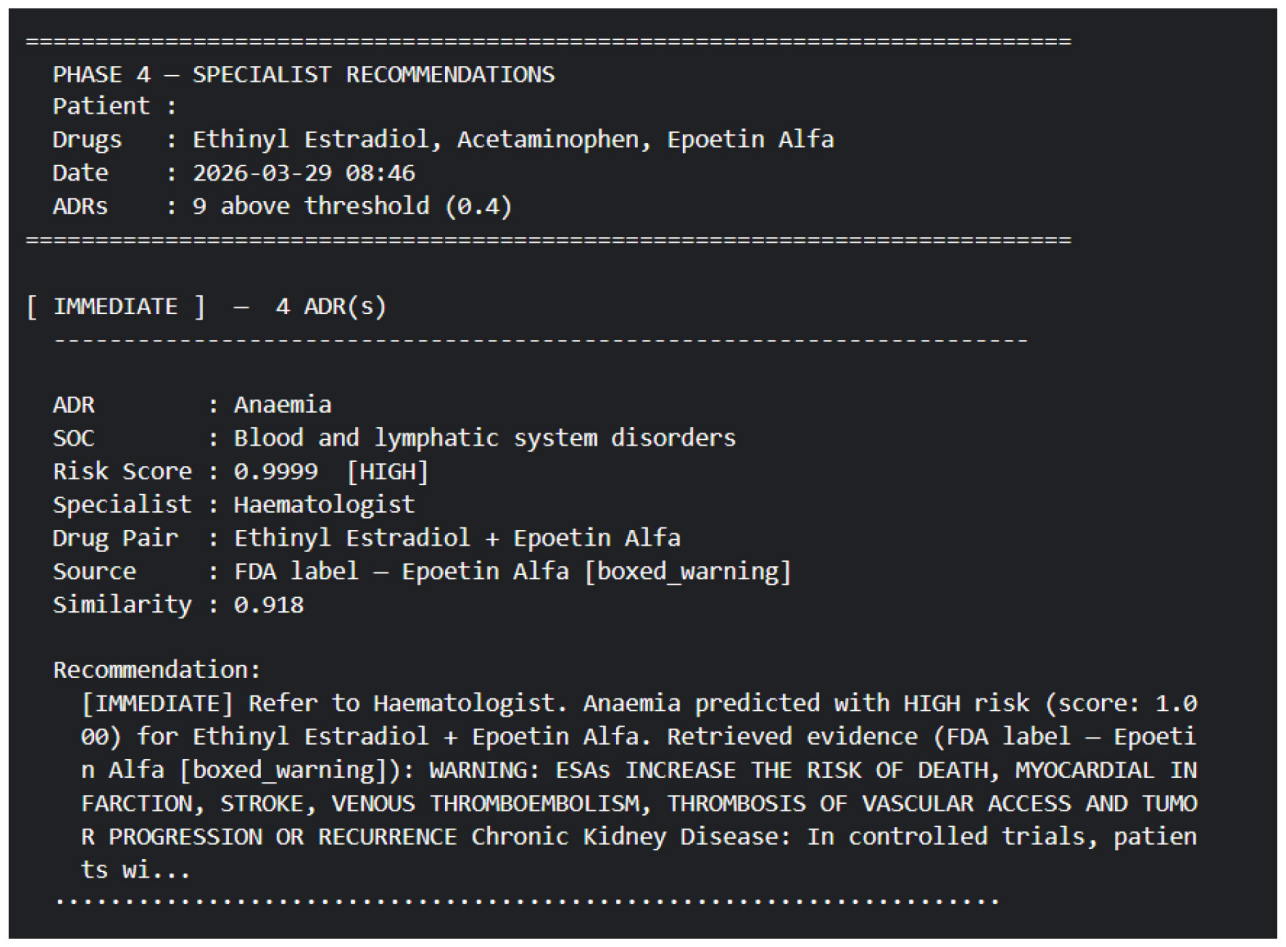
Example output on Command Line Interface (CLI) showing one of the predicted ADRs and its corresponding clinical recommendation for a given set of drugs.

Figure 8, it shows the clinical guidance and specialist recommendation for one of the predicted ADR out of the 9 predicted ADRs for the set of drugs (Ethinyl Estradiol, Acetaminophen, Epoetin Alfa).

The ADR term Anemia was assigned to the MedDRA System Organ Class (SOC), blood and lymphatic system disorders, via the SOC lookup, then, combined with the HIGH level risk score of 0.9999, an “immediate” urgency tier was assigned. The specialist routing mapped Blood and lymphatic system disorders to a hematologist, who is the clinically appropriate specialist for anemia management.

The RAG retrieval component queried the dynamically constructed FAISS vector store using the structured query built from the drug pair, ADR term, and SOC. The retrieved top chunk was the FDA boxed warning for Epoetin Alfa, which had a cosine similarity of 0.918, indicating that the retrieved document is highly relevant to the query. The highest similarity score of all 9 recommendations from the validation run supports the conclusion that the retrieval identified the most relevant clinical evidence associated with this specific ADR-drug combination.

### 4.6 Comparative analysis of the proposed DrugPred model

To evaluate the accuracy of the predicted drug interactions produced by this new model, a comparative analysis using multiple established and well-known baseline methods was utilized. Performance metrics such as AUROC and AUPRC were utilized for a consistent comparison across each method.

We have chosen benchmarks that depict diverse architectural styles in relation to predicting interactions between drugs. The first examples of these methods use tensor factorization to create representations of relation-based drug data. RESCAL Nickel et al. (2011) uses a 3-way tensor for multi-relational collective training and has an AUROC of 0.693 and an AUPRC of 0.613. While it helps lay foundational work in the area of relational learning, RESCAL's thin tensor factorization only captures the linear aspects of biochemical interactions between drugs. Similarly, DEDICOM Perros et al. (2017) used a scalable PARAFAC2-based tensor decomposition and had an AUROC of 0.705 and an AUPRC of 0.637, which represents an improvement but demonstrates how factorization-based approaches have significant limitations when modeling complex biological data through a factorization framework.

Graph-based and embedding approaches marked a considerable step forward in predictive performance. DeepWalk Perozzi et al. (2014) introduced random walk-based neural embeddings to encode structural information from drug interaction networks, yielding an AUROC of 0.761 and AUPRC of 0.737. Decagon Zitnik et al. (2018) took a more targeted approach by modeling polypharmacy side effects directly using graph convolutional networks over a multimodal graph of drugs and proteins, reaching an AUROC of 0.872 and AUPRC of 0.832. A concatenated drug features baseline derived from the same work Zitnik et al. (2018) achieved an AUROC of 0.793 and AUPRC of 0.764, demonstrating that richer feature representations, even without deep graph reasoning, offer meaningful gains over pure tensor methods.

Knowledge graph embeddings substantially improved performance with TransE Bordes et al. (2013) achieving strong AUROC rates of 0.949; DistMult Yang et al. (2015) also achieved strong AUROC (0.923) and AUPRC Rates (0.898) with this model using a bilinear diagonal formulation for knowledge base completion; KBLRN Malone et al. (2018) was specifically targeting polypharmacy adverse drug interaction side effect predictions through knowledge graph completions and achieving AUROC rates of 0.899; and ComplEx Trouillon et al. (2016) achieved the best overall performance of all of the methods we discussed previously using complex-valued embeddings to capture asymmetric relational patterns with an AUROC of 0.965 and an AUPRC of 0.944. A structured summary of all baseline results alongside the proposed model's performance is presented in Table 9.

**Table 9:** Performance comparison of the proposed model against established baseline methods on the DDI prediction task.

Approach	AUROC	AUPRC	References
Decagon	0.872	0.832	Zitnik et al., 2018
RESCAL tensor factorization	0.693	0.613	Nickel et al., 2011
DEDICOM tensor factorization	0.705	0.637	Perros et al., 2017
DeepWalk neural embeddings	0.761	0.737	Perozzi et al., 2014
Concatenated drug features	0.793	0.764	Zitnik et al., 2018
TransE	0.949	0.934	Bordes et al., 2013
DistMult	0.923	0.898	Yang et al., 2015
KBLRN	0.899	0.878	Malone et al., 2018
ComplEx	0.965	0.944	Trouillon et al., 2016
**Proposed model**	0.993	0.9882	—

Even though ComplEx and TransE have shown good results, the proposed model significantly outperforms all other baseline algorithms, achieving a 0.993 AUROC and 0.9882 AUPRC. When compared to the next best method, ComplEx Trouillon et al. (2016), our proposed architecture achieves approximately 2.8% higher AUROC performance and approximately 4.4% higher AUPRC performance. These substantial improvements indicate that the proposed architecture is much better at representing the complex, high-dimensional spaces involved in modeling drug-drug interactions than all prior models and therefore supports both the overall design of the model as well as the representational method used to define features used by the model.

### 4.7 Limitations

There are several critical constraints related to the DrugPred framework that need to be mentioned, despite its showing good potential. Firstly, this investigation utilized exclusively retrospective data from the FAERS databases (OFFSIDES and TWOSIDES). Both of these databases have been shown to have large amounts of underreported cases of mild adverse events. Furthermore, the way these cases were reported has been found to be biased toward reporting more severe and urgent events. Even though DrugPred offers labels that are based on the PRR (proportional reporting ratio), they do not indicate the presence of a causal association between the drug and the reported event; they only show a statistical association between the drug and the event.

Secondly, as of the date of this writing, the DrugPred model has not been validated against EHRs or in real-world clinical practice prior to publishing, as all validations were performed against only historical data. This restricts our ability to infer any findings regarding the drug's efficacy in a natural environment and its use clinically. The recommendation module uses external databases (OpenFDA and PubMed) to retrieve information about drug-drug interactions, but it may contain outdated information, retrieve low-quality reports, and create hallucinations due to limited relevant literature, which would reduce recommendation accuracy.

The performance numbers we reported (AUROC of 0.993 and PR-AUC of 0.988) correspond to the best-performing run out of multiple training runs conducted with different random seeds. Because training the complete model with the EdgeConv-GNN and Bio_ClinicalBERT together takes a lot of computing power, we did not compute mean performance, standard deviation, or confidence intervals across these runs, nor did we perform calibration checks such as Brier scores, which would provide stronger supporting evidence.

Currently, the system uses only 10 MedDRA System Organ Classes as inputs for creating recommendations. This limits the ability of the system to identify rare ADRs that do not fit into these well-defined classes. The system also has high processing and resource demands due to the EdgeConv-GNN and Bio_ClinicalBERT implementation, and thus, its use in resource-limited environments may be constrained. Finally, the pairwise modeling for drug-drug interactions does not account for complex interactions between drugs as seen in real-life polypharmacy patients taking multiple medications.

## 5 Conclusion

Existing studies on deep learning have attempted to model drug-drug interactions and have relied upon isolated statistical signals, and do not have the semantic depth necessary to enable actionable clinical use. In response to this, we propose a new multi-model framework utilizing EdgeConv-based Graph Neural Networks combined with Bio_ClinicalBERT, allowing for a holistic understanding of both relational and contextual ADR risks. The proposed hybrid system outperformed the benchmark systems using the same pharmacovigilance datasets by achieving a 95% accuracy and a macro-averaged F1 score of 0.95. To address the inherent black box issues of deep learning, the system utilized a 768-dimensional semantic embedding space in combination with historical associations to implement a Retrieval-Augmented Generation (RAG) model pipeline to predict possible drug-toxicity connections. Currently, the research is limited to a small set of drug-to-target interactions and patient records; to strengthen the applicability of the framework, future work will require validation on other clinical datasets from multiple sites with variable patient populations. Furthermore, the architecture's modular design will allow for integration with hospital information management systems and mobile clinical decision-support tools.

## Data Availability

The original contributions presented in the study are included in the article/supplementary material, further inquiries can be directed to the corresponding author.
